# Correction: Thermophysical Properties of Lignocellulose: A Cell-Scale Study Down to 41K

**DOI:** 10.1371/journal.pone.0122429

**Published:** 2015-03-26

**Authors:** The PLOS ONE Staff

Fig. 5 is a duplicate of Fig. 4. Please view the correct Fig. 5 here.

**Fig 5:**
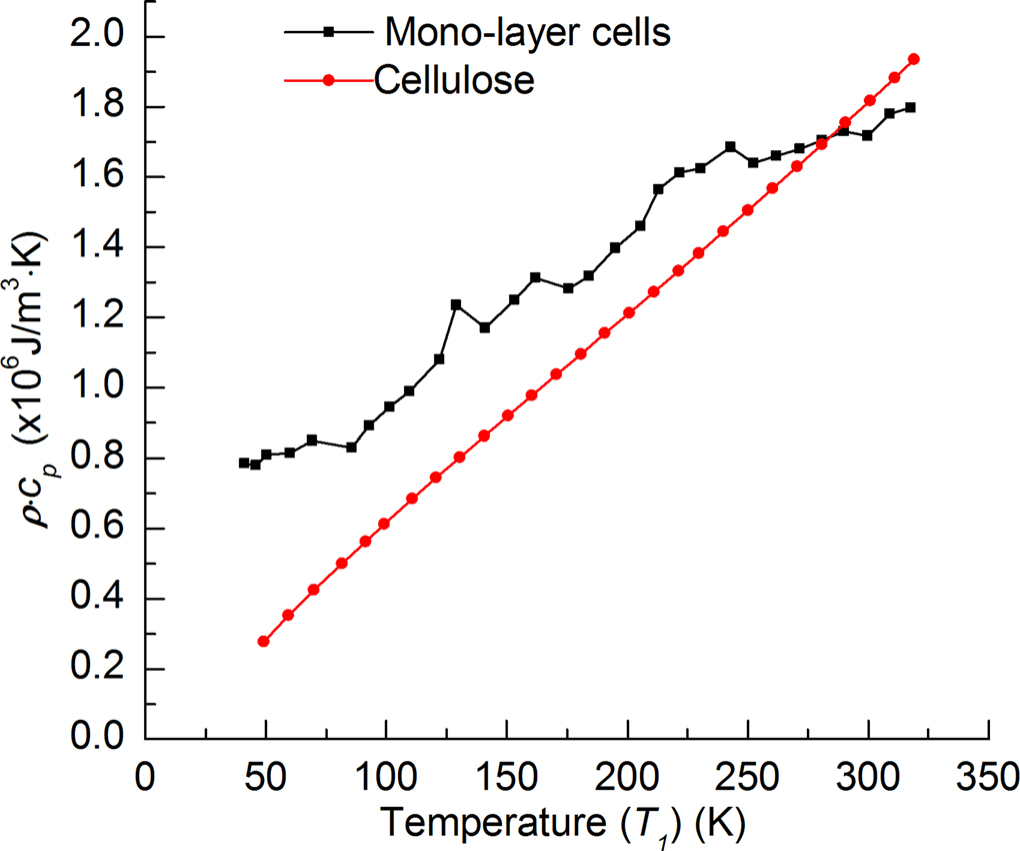
Temperature dependence of the cell-scale lignocellulose's volumetric specific heat.
